# The Effects of Home-Based Strengthening Calf Muscle Exercise Program with Graduated Compression Stockings on Disease Severity, Muscle and Joint Function, and Quality of Life Among People with Chronic Venous Insufficiency: A Randomized Controlled Trial

**DOI:** 10.3390/healthcare14081045

**Published:** 2026-04-15

**Authors:** Kulweena Sisayanarane, Suchira Chaiviboontham, Piyawan Pokpalagon, Nutsiri Kittitirapong, Chutirat Sonpee

**Affiliations:** 1 Ramathibodi School of Nursing, Faculty of Medicine Ramathibodi Hospital, Mahidol University, Bangkok 10400, Thailand; kulweena.sis@student.mahidol.ac.th (K.S.); piyawan.pok@mahidol.ac.th (P.P.); 2 Department of Surgery, Faculty of Medicine Ramathibodi Hospital, Mahidol University, Bangkok 10400, Thailand; nutsiri.kit@mahidol.ac.th; 3 Nursing Division, Ramathibodi Hospital, Faculty of Medicine Ramathibodi Hospital, Mahidol University, Bangkok 10400, Thailand; chutirat.sop@mahidol.ac.th

**Keywords:** chronic venous insufficiency, graduated compression stocking, physical exercise program, randomized controlled trial

## Abstract

**Background**: Chronic venous insufficiency (CVI) is characterized by venous dysfunction in the lower extremities, leading to increased venous pressure, edema, and reduced quality of life. **Objectives**: This study aimed to evaluate the additional effect of a structured home-based calf muscle strengthening exercise program when combined with standard compression therapy, by comparing disease severity, musculoskeletal function, and quality of life over time between patients receiving compression therapy alone and those receiving combined intervention. **Methods**: A randomized controlled trial was conducted in 50 patients with CVI (CEAP C_3_–C_5_), who were assigned to an experimental group (n = 25) and a control group (n = 25). Outcomes were assessed at baseline, week 6, and week 12. Disease severity was measured using the Revised Venous Clinical Severity Score (rVCSS), and swelling, muscle, and joint function were assessed using calf muscle strength and ankle range of motion. Quality of life outcomes were assessed using the chronic venous disease quality of life questionnaire (CIVIQ-20). Data were analyzed using two-way repeated measures ANOVA. This trial was registered retrospectively at the Thai Clinical Trials Registry (registration number: TCTR20260307002). **Results**: Significant group × time interaction effects were observed for disease severity (right leg: F = 81.562, *p* < 0.001, η^2^p = 0.630; left leg: F = 73.765, *p* < 0.001, η^2^p = 0.606), indicating greater improvement in the experimental group over time. Calf muscle strength significantly increased in the experimental group (right leg: F = 395.246, *p* < 0.001, η^2^p = 0.892; left leg: F = 87.278, *p* < 0.001, η^2^p = 0.645). Ankle range of motion also improved significantly (*p* < 0.001). Quality of life showed significant improvement with a group × time interaction effect (F = 66.104, *p* < 0.001, η^2^p = 0.579). **Conclusions**: A structured home-based calf muscle strengthening exercise program combined with compression therapy produced significant improvements in disease severity, musculoskeletal function, and quality of life over time, demonstrating an additive therapeutic effect in patients with CVI.

## 1. Introduction

Chronic venous insufficiency (CVI) is a disease characterized by venous system pathology and abnormal function, particularly in the lower limbs [1]. The severity of the disease ranges from level C_3_, which is leg swelling, to level C_6_, which is the presence of venous ulcers in the lower limbs. The standard internationally accepted classification of the clinical manifestations of chronic venous disease is the CEAP (Clinical–Etiology–Anatomy–Pathophysiology) classification [1,2,3]. Older adults have a much higher incidence of CVI than younger adults [2]. Risk factors for developing CVI include being female, prolonged standing or sitting, a family history of venous insufficiency, smoking [3], increasing age, obesity, and pregnancy [1].

Treatment approaches for CVI include surgery, medication, and conservative treatments. Conservative treatment, such as compression therapy, is widely used. External compression with stockings improves venous return efficiency and reduces swelling and other symptoms that affect blood circulation and the venous walls [4]. In addition, there are alternative exercise methods to treat CVI to increase lower limb muscle strength and ankle mobility, as well as to improve venous reflux efficiency [1]. The goal of treating CVI is to reduce the severity of symptoms and prevent complications, including venous ulcers in the legs, which have an incidence of 0.7%, and to promote venous ulcer healing [3].

In patients with CVI, studies have indicated that the use of compression stockings before, during, and after exercise helps reduce symptoms, improve venous circulation, strengthen calf muscles, enhance ankle mobility, and improve overall function and quality of life [1]. A systematic review of studies examining the effects of exercise on calf muscle contraction, muscle strength, ankle range of motion, and quality of life in patients with varying severity of CVI found that in patients with mild disease, exercise promotes venous reflux, increases muscle strength, improves ankle range of motion, and enhances quality of life [4].

Monitoring the outcomes of exercise therapy in patients with CVI primarily involves assessing the severity of symptoms and signs using standardized tools [1,2], such as the Revised Venous Clinical Severity Score (rVCSS) [5]. This tool, developed from the CEAP classification, is widely used to assess disease severity, but its effectiveness in individual patients with CVI remains limited. It is recommended that the type, level, frequency, and duration of exercise be specified, and that standardized outcome assessment tools be used in conjunction with it [2]. Another crucial aspect of assessing disease severity is edema. International studies comparing CVI patients with severe edema ranging from mild leg swelling (C_3_) to chronic venous insufficiency with healed leg ulcers (C_5_) showed that exercise significantly reduced edema more effectively than leg elevation [6].

Statistics from Ramathibodi Hospital, Bangkok, Thailand, indicate that approximately 2000–2200 patients with chronic venous insufficiency (CVI) are managed annually in home-based settings. However, adherence to treatment following discharge remains suboptimal, which may be related to limited patient awareness and self-management capacity. Despite the established benefits of both compression therapy and exercise, evidence regarding the additional effect of structured home-based exercise programs when combined with standard compression therapy remains limited, particularly in real-world clinical settings. Most previous studies have focused on either exercise interventions or compression therapy alone, with limited investigation into their combined and incremental benefits. No study has clearly evaluated the additive effect of structured home-based exercise programs combined with compression therapy using a randomized controlled design in routine clinical practice. Moreover, both compression use and exercise require patients’ ability to perform consistent self-care behaviors; however, this self-care dimension has been insufficiently addressed in prior research. Therefore, this study applies self-care theory as a conceptual framework to support patients’ capacity to adhere to and implement these interventions in daily life.

In Thailand, there is also a lack of studies evaluating the effectiveness of structured home-based exercise programs integrated into routine care for patients with CVI. Given the challenges in treatment adherence, practical and accessible interventions are needed to enhance self-management and improve clinical outcomes. Therefore, this study aimed to evaluate the additional effect of a structured home-based calf muscle strengthening exercise program when combined with standard compression therapy, by comparing disease severity, musculoskeletal function, and quality of life over time between patients receiving compression therapy alone and those receiving combined intervention. We hypothesized that patients receiving the combined intervention would demonstrate significantly greater reductions in disease severity and improvements in musculoskeletal function and quality of life over time compared to those receiving standard care alone.

## 2. Materials and Methods

### 2.1. Study Design

This study was a single-blinded, two-arm, and parallel randomized controlled trial. Participants were allocated to an experimental group (EG) and a control group. (CG). All participants were informed of the study’s purpose and provided consent before enrollment. Recruitment, intervention delivery, and follow-up assessments were conducted at the outpatient surgery unit, vascular surgery and organ transplantation clinic, Ramathibodi Hospital, Bangkok, Thailand. The study was approved by the Human Research Ethics Committee, Faculty of Medicine, Ramathibodi Hospital, Mahidol University (Identifier: MURA2024/690, approved on 26 September 2024). The trial was registered at the Thai Clinical Trials Registry (Identifier: TCTR20260307002, approved on 7 March 2026). Registration was completed retrospectively due to an error during the initial submission process. However, the ethics committee approved the study protocol and participant recruitment procedures prior to study initiation. All study procedures were strictly conducted in accordance with the approved protocol before the enrollment of participants.

Following the baseline assessment, participants received their respective intervention for 12 weeks. A maintenance program was conducted to encourage participants to continue engaging. Follow-up was conducted by phone and via a communication application (LINE application) throughout the intervention. Outcome evaluations were performed at baseline, during the 6-week intervention, and at the end of the study.

### 2.2. Sample Size

The sample size was calculated using G*Power 3.1.9.7 [7]. The researchers calculated the effect size using Cohen’s model [8], defining an effect size of 0.25 as a medium effect. The researchers used a two-way ANOVA with repeated measures, setting the significance level (α) to 0.05 and the power level to 0.80, resulting in a sample size of 20 per group. To account for potential participant dropout, the researchers increased the sample size by 20%, to 25 people per group. Therefore, this study included 50 participants, divided into an experimental group of 25 and a control group of 25.

### 2.3. Participants

The inclusion criteria were as follows: (1) aged 18 years and over, of either gender; (2) patients diagnosed with CVI who were classified in CEAP Class 3–5, as assessed by a physician specializing in vascular surgery; (3) a person with full consciousness (individuals over 60 years old were screened with the Mini-Cog [9]); (4) consent to participate in this research voluntarily; (5) able to use a telephone to communicate, and the patient or relative has the LINE application to communicate; (6) not diagnosed with deep vein thrombosis in both legs based on the results of a duplex ultrasound within the past 3 months; and (7) no clinical signs on initial physical examination, including unilateral leg swelling (difference of more than 3 cm), red legs, tenderness, tightness, swollen veins, warm skin at the site of a blood clot, or a positive Homan’s sign. The exclusion criteria included (1) severe or unstable cardiopulmonary pathology; (2) unable to stand, having problems with strength or balance, at risk of falls; and (3) thrombophlebitis or open wounds on the legs that do not allow the wearing of medical stockings, or who have severe leg swelling preventing the use of medical stockings.

### 2.4. Randomization and Blinding

#### 2.4.1. Randomization and Allocation Concealment

Participants were randomly assigned to the experimental or control group using a computer-generated randomization sequence. Allocation concealment was ensured using sequentially numbered, sealed, opaque envelopes prepared by an independent individual not involved in the study. Each envelope contained the group assignment according to the randomization sequence and was opened only after participant enrollment, ensuring that group allocation could not be predicted in advance.

#### 2.4.2. Blinding

Due to the nature of the intervention, participants could not be fully blinded. However, outcome assessors were blinded to group allocation. Independent assessors who were not involved in the intervention delivery or group assignment conducted all outcome measurements.

#### 2.4.3. Maintenance of Blinding During Outcome Assessment

Outcome assessments were conducted in separate settings to minimize interaction between groups, and assessors were not informed of participants’ group assignments throughout the study period.

### 2.5. Intervention

#### 2.5.1. The Experimental Group

The exercise program consisted of four components designed to improve calf muscle strength and ankle joint mobility. All exercises combined stretching and weight-bearing activities. (1) Ankle mobility enhancement exercises: Participants were seated on a chair with back support, knees flexed at approximately 90 degrees, and feet placed flat on the floor. They performed alternating ankle dorsiflexion and plantarflexion movements. One repetition included both movements. Participants completed 12 repetitions per set, for a total of 5 sets, with 60 s of rest between sets. (2) Calf muscle strengthening exercises: Participants stood upright with feet flat on the floor and slowly raised their heels to stand on their toes. The position was held for 5 s before lowering the heels. Participants performed 10 repetitions per set, for a total of 3 sets, with 60 s of rest between sets. (3) Calf stretching exercise (Position 1): Participants stood facing a wall at a distance of approximately 1–2 feet, with both hands placed on the wall and arms extended. They leaned forward while keeping the heels on the ground until a mild stretch was felt in the posterior calf muscles. The position was held for 30 s and repeated 5 times, with 10 s of rest between repetitions. (4) Calf stretching exercise (Position 2): Participants stood facing a wall, with one foot positioned behind the other. The back leg remained straight with the heel on the ground, while the front knee was slightly flexed. The stretch was held for 30 s and repeated for both legs. Each set consisted of 3 repetitions per leg, for a total of 3 sets, with 10 s of rest between repetitions.

Participants received initial education sessions conducted by the researcher in small groups (≥2 participants), including information on CVI, treatment approaches, and the benefits of exercise and compression therapy. The researcher demonstrated all exercises, and participants were required to perform return demonstrations to ensure correct technique prior to starting the program. Ongoing supervision and support were provided through scheduled follow-ups via telephone and the LINE application at weeks 2, 4, 6, 8, 10, and 12. Participants were provided with exercise manuals and instructional videos, which were accessible at any time. They were also able to contact the researcher for guidance if needed. To ensure adherence and correct performance, participants recorded their exercise activities in a structured self-assessment tracking form. The researcher monitored adherence and provided reminders, feedback, and problem-solving support throughout the intervention period.

Outcome measures were assessed at three time points: baseline (week 1), mid-intervention (week 6), and immediately after completion of the program (week 12).

#### 2.5.2. The Control Group

The control group received standard care, including medication and medical stockings as per the doctor’s treatment plan, as much as possible during the day. The level of compression applied by the stockings is moderate compression between 20 and 30 mmHg. They only removed the stockings at night and then used pillows to elevate their legs above their hearts.

### 2.6. Outcome Measurements

#### 2.6.1. Disease Severity

Disease severity was assessed using the Revised Venous Clinical Severity Score (rVCSS) [5]. The instrument was translated into Thai using a forward–backward translation process by bilingual experts. Content validity was evaluated by three experts in vascular surgery, nursing, and physiotherapy, yielding a content validity index (CVI) of 0.89. The reliability of the Thai version was tested in a pilot sample of 10 patients with similar characteristics to the study population, resulting in a Cronbach’s alpha coefficient of 0.70.

Edema was assessed using a structured edema record developed based on the previous literature. Leg circumference was measured in centimeters using a non-elastic measuring tape, starting at the first metatarsophalangeal joint and continuing at 10 cm intervals up to 50 cm. Measurements were taken for both legs and averaged. Content validity of the edema assessment tool was confirmed by three experts (CVI = 0.89), and reliability testing demonstrated high internal consistency (Cronbach’s alpha = 0.97).

#### 2.6.2. Muscle and Joint Function

Calf muscle strength was assessed using a digital handheld dynamometer (microFET^®^2, Hoggan Scientific, LLC, Salt Lake City, UT, USA), which was calibrated according to the manufacturer’s recommendations and used consistently throughout the study period. Participants were positioned in a prone position on a bed, with hips and knees fully extended and ankles maintained at a neutral position (90°). A transducer pad was placed at the proximal metatarsal region. Participants were instructed to perform maximal plantar flexion against resistance for approximately 5 s [10,11,12,13,14]. Measurements were recorded in kilogram-force (kgf). Each leg was tested separately, with three repeated measurements at 15 s intervals. The mean value of the three trials was used for analysis. All assessments were conducted at baseline, week 6, and week 12 by trained assessors following standardized procedures.

The calf muscle strength assessment form was developed based on a review of the relevant literature. Content validity was evaluated by three experts in vascular surgery, nursing, and physiotherapy, yielding a content validity index (CVI) of 0.89. Reliability testing in a pilot sample of 10 patients demonstrated acceptable internal consistency (Cronbach’s alpha = 0.80). The handheld dynamometer is a widely used instrument with established reliability and validity for measuring lower limb muscle strength in clinical and research settings.

Ankle range of motion (ROM) was measured using a universal goniometer, a widely accepted and reliable instrument for assessing joint mobility in clinical settings. The same device was used throughout the study period to ensure measurement consistency. Participants were seated on a bed with their feet positioned parallel to the floor. The stationary arm of the goniometer was aligned with the anteromedial aspect of the tibia, the fulcrum was placed over the medial malleolus, and the movable arm was aligned with the first metatarsal. Participants were instructed to actively perform maximal plantar flexion and dorsiflexion [10,12,14,15,16]. ROM was recorded in degrees for both plantar flexion and dorsiflexion. Each measurement was repeated three times for each position, and the mean value was used for analysis. Measurements were obtained for both legs at baseline, week 6, and week 12 by trained assessors following standardized procedures.

The ankle ROM assessment form was developed based on a review of the relevant literature. Content validity was evaluated by three experts in vascular surgery, nursing, and physiotherapy, yielding a content validity index (CVI) of 0.89. Reliability testing in a pilot sample of 10 patients demonstrated acceptable internal consistency (Cronbach’s alpha = 0.80). Prior to the study, the measurement procedure was pilot-tested to ensure feasibility and consistency.

#### 2.6.3. Quality of Life

Quality of life was assessed using the Chronic Venous Disease Quality of Life Questionnaire (CIVIQ-20) [17]. The Thai version of the questionnaire consists of 20 items across four domains: physical (4 items), psychological (9 items), social (3 items), and pain (4 items). Content validity of the instrument was evaluated by three experts in vascular surgery, nursing, and physiotherapy, yielding a content validity index (CVI) of 0.89. The questionnaire was reviewed and refined based on expert recommendations prior to use. Reliability testing was conducted in a pilot sample of 10 patients with characteristics similar to the study population, demonstrating excellent internal consistency (Cronbach’s alpha = 0.94). The CIVIQ-20 is a widely used and validated instrument for assessing quality of life in patients with chronic venous insufficiency. All assessments were conducted at baseline, week 6, and week 12 using standardized procedures.

### 2.7. Statistical Analysis

Baseline demographic and clinical characteristics of the experimental and control groups were analyzed using descriptive statistics, including frequency, percentage, mean, standard deviation, and range. Descriptive results are reported as means with 95% confidence intervals and standard deviations.

Changes in disease severity (rVCSS), bilateral leg circumference, calf muscle strength, ankle range of motion, and quality of life (CIVIQ-20) over time were analyzed using two-way ANOVA with repeated measures to examine the main effects of group, time, and group × time interaction. Prior to analysis, key statistical assumptions were evaluated and met, including normality (Shapiro–Wilk test), homogeneity of variance (Levene’s test), and homogeneity of covariance matrices (Box’s test). The assumption of sphericity was assessed using Mauchly’s test; the results indicated that sphericity was satisfied only for ankle range of motion in plantar flexion, whereas it was violated for all other variables. Accordingly, the Greenhouse–Geisser correction was applied to adjust for violations of sphericity. When significant main or interaction effects were identified, Bonferroni-adjusted post hoc pairwise comparisons were performed to examine differences between baseline, week 6, and week 12. The observed statistical power for the primary disease severity and quality-of-life group × time interaction outcomes ranged from 0.98 to >0.99, supporting the adequacy of the final sample size.

## 3. Results

### 3.1. Background Information of Participants

**Figure 1:**
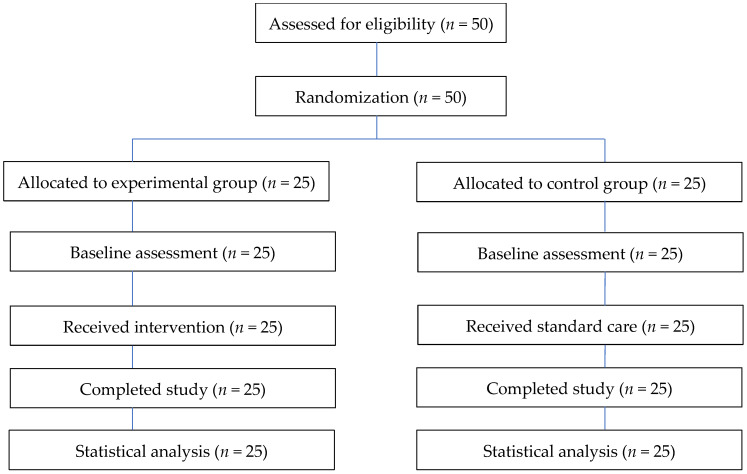
Participant flow diagram of the study.

Fifty participants were randomly allocated to the experimental group (n = 25) and the control group (n = 25). The 50 participants all completed the study (Figure 1).

#### 3.1.1. Demographic

The experimental group consisted mostly of females (72%), with an average age of 69.64 years (SD = 11.28). In total, 36% had a Body Mass Index (BMI) between 25.0 and 29.9 kg/m^2^, 40% were unemployed, 44% exercised 2–3 days a week for at least 30 min each day, 68% stood or sat for less than 4 h a day at work, 72% had never smoked, and 44% wore stockings almost all day, as shown in Table 1.

The control group was predominantly female (64%), with an average age of 66.40 years (SD = 9.74). In total, 44% had a Body Mass Index (BMI) greater than 30 kg/m^2^, 48% were unemployed, 48% did not exercise at all, 52% stood or sat for more than 4 h per day at work, 88% had never smoked, and 48% did not wear stockings, as shown in Table 1.

Baseline demographic and clinical characteristics were generally balanced between groups, except for regular compression stocking use, which differed significantly at baseline (*p* = 0.046). Specifically, a higher proportion of participants in the control group reported no regular stocking use. This imbalance represents a potential confounding factor and should be considered when interpreting the longitudinal comparative outcomes.

#### 3.1.2. Past Illnesses and Treatments

The experimental group had varying disease severity in the right and left legs, with the majority (32%) having C_3_ severity in both legs. The duration of diagnosis of chronic venous insufficiency of the leg was mainly 5 years or more (52%). The majority had hypertension (56%), and 100% did not have peripheral arterial occlusion. There was no family history of venous disease (80%), and 100% had no history of fractures requiring casting or surgery on the affected leg, as shown in Table 2.

The control group had varying degrees of right and left leg disease severity, with the majority (C_3_ and C_4a_) at levels 24%. The majority (56%) had a diagnosis of chronic venous insufficiency of the leg for less than 5 years. Most had hypertension (68%), and 100% did not have peripheral arterial occlusion. A total of 60% had no family history of venous disease, and 100% had no history of fractures requiring casting or amputation of the affected leg, as shown in Table 2.

The study results showed that the characteristics of the experimental and control groups were similar, including disease severity, duration of diagnosis, comorbidities, and family history of venous disease.

Mean values with corresponding 95% confidence intervals (CIs) for all primary outcomes at baseline, week 6, and week 12 are summarized in Table 3, enhancing the precision and interpretability of the findings.

### 3.2. Disease Severity Includes the Severity of the Disease and Swelling

#### 3.2.1. The Severity of the Disease

The mean severity scores for right leg disease in the experimental and control groups did not differ significantly (F = 0.226, *p* > 0.05). The mean severity scores for left leg disease in the experimental and control groups did not differ significantly (F = 1.380, *p* > 0.05), as shown in Table 3.

#### 3.2.2. Swelling

The mean scores for right leg edema differed significantly between the experimental and control groups (F = 9.128, *p* < 0.05). The mean scores for left leg edema differed significantly between the experimental and control groups (F = 12.009, *p* < 0.05), as shown in Table 3.

### 3.3. Muscle and Joint Function Is Assessed, Including Calf Muscle Strength and Ankle Range of Motion in Plantar Flexion and Dorsiflexion

#### 3.3.1. Calf Muscle Strength

The mean scores for right calf muscle strength in the experimental and control groups differed significantly (F = 204.100, *p* < 0.05). The mean scores for left calf muscle strength in the experimental and control groups differed significantly (F = 125.918, *p* < 0.05), as shown in Table 3.

#### 3.3.2. Ankle Range of Motion in Plantar Flexion and Dorsiflexion

The mean range of motion scores for right plantar flexion ankles in the experimental and control groups differed significantly (F = 5.078, *p* < 0.05). The mean range of motion scores for right ankle dorsiflexion differed significantly between the experimental and control groups (F = 64.252, *p* < 0.05).

The mean range of motion scores for left plantar flexion ankle in the experimental and control groups did not differ significantly (F = 1.349, *p* > 0.05). The mean range of motion scores for left ankle dorsiflexion in the experimental and control groups differed significantly (F = 294.156, *p* < 0.05), as shown in Table 3.

### 3.4. Quality of Life

The mean quality of life scores of the experimental and control groups differed significantly (F = 6.860, *p* < 0.05), as shown in Table 3.

## 4. Discussion

All participants in this study were patients with chronic venous insufficiency (CVI), with the majority being female. The participants ranged in age from 31 to 86 years, with a mean age of 68.02 years (SD = 10.557). Increased age and higher disease severity have been associated with poorer quality of life [18]. Most participants presented with C_3_ severity in both legs, which is consistent with international reports indicating that C_3_–C_6_ stages account for a substantial proportion of CVI cases, with C_3_ being the most prevalent [19]. Clinical manifestations at this stage commonly include leg edema, which may lead to pain and progressive disease severity according to the CEAP classification. Hypertension was the most common comorbidity among participants. This finding is consistent with previous studies showing that age, female sex, hypertension, obesity, and smoking are significant risk factors for CVI and are associated with increased cardiovascular morbidity and mortality [19].

A baseline imbalance in compression stocking use was observed between the experimental and control groups, with a higher proportion of participants in the control group reporting no use of compression stockings at baseline. Given that compression therapy is a key component of standard care for CVI, this difference may have influenced the study outcomes and should be considered when interpreting the results. Specifically, participants in the control group may have started from a relatively lower level of therapeutic management, which could have contributed to differences observed over time.

Although both groups received compression therapy during the study period, the initial imbalance may still represent a potential confounding factor. Therefore, the findings of this study should be interpreted with caution. Future studies using randomized designs with stricter baseline equivalence or incorporating statistical adjustment for baseline differences are recommended to better isolate the effects of the intervention.

### 4.1. Disease Severity

The progressive reduction in disease severity observed over time in the experimental group may reflect the additive benefit of combining a structured home-based calf muscle strengthening program with compression therapy. Rather than indicating a cross-sectional difference at a single time point, the significant group × time interaction suggests that the intervention was associated with a more favorable longitudinal trajectory compared with standard care alone. A plausible explanation lies in the complementary physiological mechanisms of the two interventions. Compression therapy facilitates venous return and reduces venous hypertension, whereas calf muscle strengthening enhances calf pump efficiency, improves ankle mobility, and promotes lower-limb blood flow. Together, these mechanisms may reduce venous stasis and edema, thereby contributing to lower disease severity over time. This interpretation is consistent with previous evidence supporting conservative management as the first-line approach for chronic venous insufficiency, particularly the combined use of compression therapy and exercise to improve venous hemodynamics and symptom control [2,14]. Previous studies have also shown that aerobic and strengthening exercises can improve muscle performance and venous reflux efficiency, which may indirectly contribute to reduced clinical severity [11,20]. In addition, the structured and supportive nature of the home-based program—including education, supervised return demonstration, scheduled follow-up, and accessible instructional materials—may have enhanced adherence and self-management behaviors. This is conceptually aligned with Orem’s self-care theory, in which supportive–educative systems strengthen patients’ capacity to maintain therapeutic behaviors over time. Although the magnitude of improvement suggests potential clinical relevance, this interpretation should remain cautious given the relatively small sample size and baseline imbalance in compression stocking use. Larger trials with longer follow-up are warranted to confirm the durability and clinical significance of these effects.

### 4.2. Swelling

The gradual decrease in lower-extremity swelling observed in the experimental group may reflect the additive effect of combining calf muscle strengthening exercise with compression therapy over time. The significant group × time interaction suggests that the longitudinal pattern of change differed between groups, with greater and more consistent reductions in swelling observed in the intervention group. A likely explanation is the synergistic physiological effect of the two treatment components. Repeated calf muscle contractions improve the efficiency of the calf muscle pump, thereby enhancing venous return and reducing venous stasis. When combined with compression therapy, which provides sustained external pressure to facilitate venous blood flow and limit fluid accumulation, these mechanisms may contribute to progressive reductions in leg swelling and improved vascular function. The structured design of the intervention may also have supported these outcomes. Education, exercise demonstration, and regular follow-up likely enhanced adherence and self-management behaviors, while the home-based format reduced barriers to participation and promoted sustained engagement throughout the 12-week period. These findings are consistent with previous studies reporting that exercise combined with compression therapy can reduce lower-limb edema and improve symptom control in patients with chronic venous insufficiency [12]. The present study extends this evidence by demonstrating these benefits within a structured home-based program and emphasizing the importance of longitudinal change rather than isolated between-group comparisons. Although the magnitude of swelling reduction suggests potential clinical relevance, the findings should be interpreted cautiously given the relatively small sample size, variability in adherence, and baseline imbalance in compression stocking use. Further studies with larger sample sizes and longer follow-up periods are needed to confirm the long-term sustainability of these effects.

### 4.3. Muscle and Joint Function

Incremental gains in calf muscle strength and ankle range of motion observed in the experimental group may reflect the additive benefit of combining a structured home-based exercise program with compression therapy over time. The significant group × time interaction supports the interpretation that the intervention was associated with a more favorable longitudinal trajectory compared with standard care alone. A physiologically plausible explanation is that repeated calf muscle strengthening enhances the efficiency of the calf muscle pump, thereby promoting venous return and improving lower-limb circulation. In parallel, repetitive ankle movements, including plantar flexion and dorsiflexion, may improve joint mobility, flexibility, and functional movement patterns. These musculoskeletal adaptations may help reduce venous stasis and support better lower-extremity function in patients with chronic venous insufficiency. When combined with compression therapy, which provides sustained external pressure to facilitate venous blood flow, these mechanisms may act synergistically to improve muscle performance and ankle mobility over time. This pattern supports the concept of an additive longitudinal effect rather than immediate differences between groups at isolated time points. The structured and supportive design of the intervention may also have contributed to these improvements. Education, exercise demonstration, and continuous follow-up likely enhanced adherence and long-term engagement, while the home-based format reduced barriers to participation and supported sustained behavioral change throughout the 12-week period.

These findings are consistent with previous studies showing that exercise combined with compression therapy can improve muscle strength and ankle mobility in patients with chronic venous insufficiency [14]. The present study further extends this evidence by demonstrating these benefits within a structured home-based self-management program and by emphasizing the importance of temporal change patterns. Although the magnitude of improvement suggests potential clinical relevance, the findings should be interpreted cautiously in light of the relatively small sample size, variability in adherence, and baseline imbalance in compression stocking use. Larger studies with longer follow-up are needed to confirm the durability and clinical significance of these functional improvements.

### 4.4. Quality of Life

The quality of life showed greater improvements over time in the experimental group, which may reflect the additive effects of combining calf muscle strengthening exercises with compression therapy in a structured home-based program. The significant group × time interaction indicates a more favorable longitudinal trajectory compared with standard care alone. A clinically meaningful explanation is that reductions in symptom burden—particularly leg swelling, discomfort, and functional limitation—may have improved participants’ ability to perform daily activities and maintain mobility. Improvements in calf muscle strength and ankle mobility may also have enhanced physical functioning, which likely contributed to better patient-reported outcomes. The magnitude of change in CIVIQ-20 scores (approximately 12–13 points), together with the large effect sizes observed (η^2^p > 0.50), suggests potential clinical relevance beyond statistical significance. The structured and supportive design of the intervention may have further strengthened these benefits. Education, return demonstration, continuous follow-up, and accessible home-based materials likely improved adherence, confidence in self-management, and sustained engagement throughout the 12-week period. These factors may also have contributed to psychological and social benefits, including reduced anxiety and greater participation in daily activities. These findings are consistent with previous studies reporting that exercise combined with compression therapy can improve quality of life in patients with chronic venous insufficiency [10,21,22]. The present study extends this evidence by demonstrating these effects within a structured home-based self-management program and by emphasizing the importance of longitudinal change patterns rather than isolated between-group comparisons. Nevertheless, the findings should be interpreted cautiously, given the relatively small sample size, baseline differences in compression stocking use, and potential confounding effects of pre-existing exercise behavior. Future studies with larger samples, longer follow-up, and stricter control of baseline physical activity are warranted to confirm the durability and clinical significance of these quality-of-life improvements.

Importantly, these improvements may also be clinically meaningful. Reduced swelling and disease severity may improve mobility and physical comfort, while gains in calf muscle strength and ankle mobility may enhance venous return and lower-limb function. Furthermore, the approximately 12–13-point improvement in CIVIQ-20 scores suggests a meaningful benefit in patient-reported quality of life and daily functioning.

## 5. Conclusions

This study suggests that a structured home-based calf muscle strengthening exercise program, when combined with medical compression therapy, may be associated with additional improvements over time in reducing disease severity, improving ankle range of motion, and enhancing quality of life in patients with chronic venous insufficiency. However, these findings should be interpreted with caution, as they remain preliminary due to the study design and baseline differences in compression stocking adherence between groups, which may have acted as a potential confounding factor. Therefore, further well-designed randomized controlled trials with larger sample sizes and more rigorous control of baseline characteristics are needed to confirm the independent and long-term effects of the intervention. From a clinical perspective, this program may serve as a practical and low-cost approach that nurses can incorporate into routine care by providing education, guidance, and support to patients. In addition, the findings may be useful for teaching nursing students and newly graduated nurses in the management of patients with chronic venous insufficiency.

### 5.1. Limitations of the Study

This study has several important limitations. First, the relatively small sample size may have limited statistical power and reduced the generalizability of the findings. Second, the retrospective trial registration may introduce a potential risk of reporting bias. Third, because both groups received compression therapy, the two-group design did not allow complete separation of the independent effects of the exercise program from those of compression therapy. In addition, a baseline imbalance in regular compression stocking use and pre-existing exercise behaviors may have acted as potential confounding factors. Although baseline exercise frequency did not differ significantly between groups, habitual physical activity may still have influenced outcomes by improving venous return and calf muscle pump function. Participant blinding was not feasible due to the nature of the intervention, introducing a possible risk of performance bias. Variability in follow-up procedures and limited outcome monitoring may also have affected the consistency of repeated assessments.

Finally, the inclusion criteria were restricted to patients with CVI severity levels C_3_–C_3_, which may limit the applicability of the findings to patients with milder or more advanced disease stages. Therefore, the findings should be interpreted with caution. Future studies with larger sample sizes, prospective trial registration, stricter control of baseline physical activity, and more rigorous study designs are recommended to confirm these findings.

### 5.2. Implications for Clinical Practice

Despite these limitations, the findings suggest that elderly individuals can maintain high adherence to structured care programs when appropriate follow-up and support are provided. The need for supplementary telephone follow-up highlights the importance of using multiple communication channels, rather than relying solely on digital platforms, when caring for older adults. Additionally, coordination across different healthcare systems to facilitate follow-up appointments may reduce patient burden and enhance access to care. Clinicians should consider flexible follow-up strategies and integrated care pathways, particularly for patients with CVI at moderate severity levels, while exercising caution when applying these results to other CVI populations.

## Figures and Tables

**Table 1:** Demographic of patients with chronic venous insufficiency in the experimental (n = 25) and control groups (n = 25).

Demographic	Participants				Chi-Square
	The Experimental Group		The Control Group		
	Number	Percentage	Number	Percentage	
Gender					
Male	7	28	9	36	0.762
Female	18	72	16	64	
Age (Year) Min–Max = 31–86 years, Mean = 68.02, SD = 10.56, *p*-value = 0.299					
30–39	1	4	-	-	
50–59	3	12	2	8	
60–69	7	28	1	4	
70–79	12	48	12	48	
80–89	2	8	9	36	
Body Mass Index (BMI) (kg/m^2^)					
18.50–22.90	8	32	7	28	0.481
23.00–24.90	2	8	1	4	
25.00–29.90	9	36	6	24	
More than 30.00	6	24	11	44	
Exercise plan					
Do it every day for 30 min	6	24	7	28	0.309
2–3 days a week, at least 30 min a day	11	44	6	24	
Do not do it at all	8	32	12	48	
Lifestyle patterns					
Standing/sitting for more than 4 h/day	8	32	13	52	0.252
Standing/sitting for less than 4 h/day	17	68	12	48	
Smoking history					
Never	18	72	22	88	0.289
Quit	7	28	3	12	
Wearing medical stockings					
Not wearing stockings	4	16	12	48	0.046 *
Wearing stockings occasionally, irregularly	10	40	5	20	
Wearing stockings almost all day	11	44	8	32	

* *p* < 0.05, BMI = Body Mass Index.

**Table 2:** Past illnesses and treatments of patients with chronic venous insufficiency in the experimental (n = 25) and control groups (n = 25).

Past Illnesses and Treatments	Participants				Chi-Square
	The Experimental Group		The Control Group		
	Number	Percentage	Number	Percentage	
Severity level of chronic venous insufficiency in the right leg					
C_3_	8	32	6	24	0.422
C_4_	4	16	4	16	
C_4a_	4	16	6	24	
C_4b_	1	4	5	20	
C_4c_	7	28	3	12	
C_5_	1	4	1	4	
Severity level of chronic venous insufficiency in the left leg					
C_3_	8	32	6	24	0.422
C_4_	4	16	4	16	
C_4a_	4	16	6	24	
C_4b_	1	4	5	20	
C_4c_	7	28	3	12	
C_5_	1	4	1	4	
Duration of time since diagnosis of chronic venous insufficiency					
Greater than or equal to 5 years	13	52	11	44	0.777
Less than 5 years	12	48	14	56	
Congenital disease *					
Diabetes Mellitus	5	20	8	32	0.519
Hypertension	14	56	17	68	0.560
Heart disease	4	16	1	4	0.346
Cerebrovascular	2	8	-	-	0.149
Rheumatoid arthritis	-	-	-	-	
Peripheral arterial occlusion	-	-	-	-	
Dyslipidemia	13	52	13	52	1.000
Obesity	-	-	1	4	0.312
Osteoarthritis	-	-	1	4	0.312
Family medical history related to venous disease					
Varicose veins	1	4	5	20	0.137
Deep vein thrombosis	-	-	2	8	
Chronic venous insufficiency	4	16	3	12	
History of a fracture that has been splinted or had surgery on the affected leg, or symptoms of venous disease	-	-	-	-	

C_3_–C_5_ = clinical severity classification according to CEAP. * More than one answer is possible.

**Table 3:** The differences in mean disease severity scores, musculoskeletal function, and quality of life were compared between the experimental (n = 25) and control groups (n = 25) using two-way repeated measure ANOVA.

Variable	Participants				F	Time	Group	Time × Group
	The Experimental Group		The Control Group					
	$\bar{X}$ (95% CI)	SD	$\bar{X}$	SD		(F, *p*-Value, η^2^p)	(F, *p*-Value, η^2^p)	(F, *p*-Value, η^2^p)
Severity of disease in the right leg						85.967; <0.001; 0.642	0.226; 0.636; 0.005	81.562; <0.001; 0.630
Before the program	7.960 ^a^ (7.123–8.797)	0.416	6.720 ^a^ (5.883–7.557)	0.416	0.226			
During the program, week 6	6.480 ^b^ (5.624–7.336)	0.426	6.720 ^a^ (5.864–7.576)	0.426				
End of program, week 12	4.880 ^c^ (4.114–5.646)	0.381	6.680 ^a^ (5.914–7.446)	0.381				
	F = 85.353 *p* < 0.001		F = 1.000 *p* = 0.327					
Severity of disease in the left leg						77.578; <0.001; 0.618	1.380; 0.246; 0.028	73.765; <0.001; 0.606
Before the program	7.880 ^a^ (6.985–8.775)	0.445	6.960 ^a^ (6.065–7.855)	0.445	1.380			
During the program, week 6	6.320 ^b^ (5.482–7.158)	0.417	6.960 ^a^ (6.122–7.798)	0.417				
End of program, week 12	4.680 ^c^ (3.966–5.394)	0.355	6.920 ^a^ (6.206–7.634)	0.355				
	F = 76.867 *p* < 0.001		F = 1.000 *p* = 0.327					
Swelling in the right leg						129.993; <0.001; 0.730	9.128; 0.004; 0.160	105.680; <0.001; 0.688
Before the program	27.680 ^a^ (26.407–28.953)	0.633	28.528 ^a^ (27.255–29.801)	0.633	9.128 *			
During the program, week 6	25.896 ^b^ (24.618–27.174)	0.636	28.414 ^a^ (27.136–29.691)	0.636				
End of program, week 12	23.512 ^c^ (22.164–24.860)	0.670	28.310 ^a^ (26.963–29.658)	0.670				
	F = 120.395 *p* < 0.001		F = 13.325 *p* < 0.001					
Swelling in the left leg						111.736; <0.001; 0.700	12.009; 0.001; 0.200	91.435; <0.001; 0.656
Before the program	27.692 ^a^ (26.421–28.963)	0.632	28.969 ^a^ (27.697–30.240)	0.632	12.009 *			
During the program, week 6	25.948 ^b^ (24.654–27.242)	0.644	28.822 ^a^ (27.528–30.116)	0.644				
End of program, week 12	23.576 ^c^ (22.248–24.904)	0.661	28.757 ^a^ (27.429–30.085)	0.661				
	F = 107.463 *p* < 0.001		F = 4.888 *p* = 0.012					
Muscle strength in the right leg						402.921, <0.001, 0.894	204.100, <0.001, 0.810	395.246, <0.001, 0.892
Before the program	17.436 ^a^ (16.816–18.056)	0.308	18.179 ^a^ (17.559–18.799)	0.308	204.100 *			
During the program, week 6	22.215 ^b^ (21.471–22.959)	0.370	18.072 ^a^ (17.328–18.816)	0.370				
End of program, week 12	35.756 ^c^ (34.563–36.948)	0.593	18.231 ^a^ (17.039–19.424)	0.593				
	F = 409.317 *p* < 0.001		F = 1.160 *p* = 0.308					
Muscle strength in the left leg						84.478, <0.001, 0.638	125.918, <0.001, 0.724	87.278, <0.001, 0.645
Before the program	19.753 ^a^ (18.978–20.528)	0.385	17.427 ^a^ (16.652–18.202)	0.385	125.918 *			
During the program, week 6	24.946 ^b^ (23.956–25.936)	0.492	17.589 ^a^ (16.599–18.579)	0.492				
End of program, week 12	33.179 ^c^ (31.164–35.195)	1.002	17.346 ^a^ (15.331–19.362)	1.002				
	F = 36.708 *p* < 0.001		F = 211.646 *p* < 0.001					
Range of motion in the right leg Plantar flexion position						195.565, <0.001, 0.803	5.078, 0.029, 0.096	186.769, <0.001, 0.796
Before the program	78.800 ^a^ (64.708–92.892)	7.009	89.640 ^a^ (75.548–103.732)	7.009	5.078 *			
During the program, week 6	108.000 ^b^ (92.895–123.105)	7.513	90.160 ^a^ (75.055–105.265)	7.513				
End of program, week 12	150.000 ^c^ (136.307–163.693)	6.810	90.480 ^a^ (76.787–104.173)	6.810				
	F = 193.182 *p* < 0.001		F = 2.537 *p* = 0.120					
Dorsiflexion position						185.715, <0.001, 0.795	64.252, <0.001, 0.572	187.168, <0.001, 0.796
Before the program	246.960 ^a^ (232.657–261.263)	7.114	206.720 ^a^ (192.417–221.023)	7.114	64.252 *			
During the program, week 6	287.760 ^b^ (272.686–302.834)	7.497	206.400 ^a^ (191.326–221.474)	7.497				
End of program, week 12	328.800 ^c^ (313.701–343.899)	7.510	206.560 ^a^ (191.461–221.659)	7.510				
	F = 186.972 *p* < 0.001		F = 1.000 *p* = 0.355					
Range of motion in the left leg Plantar flexion position						232.522, <0.001, 0.829	1.349, 0.251, 0.027	229.158, <0.001, 0.827
Before the program	71.240 ^a^ (58.353–84.127)	6.409	91.840 ^a^ (78.953–104.727)	6.409	1.349			
During the program, week 6	101.200 ^b^ (87.230–115.170)	6.948	92.080 ^a^ (78.110–106.050)	6.948				
End of program, week 12	135.520 ^c^ (122.602–148.438)	6.425	92.080 ^a^ (79.162–104.998)	6.425				
	F = 231.368 *p* < 0.001		F = 1.862 *p* = 0.176					
Dorsiflexion position						138.439, <0.001, 0.743	294.156, <0.001, 0.860	137.833, <0.001, 0.742
Before the program	243.360 ^a^ (233.309–253.411)	4.999	156.560 ^a^ (146.509–166.611)	4.999	294.156 *			
During the program, week 6	278.400 ^b^ (266.951–289.849)	5.694	156.640 ^a^ (145.191–168.089)	5.694				
End of program, week 12	315.600 ^c^ (304.854–326.346)	5.345	156.640 ^a^ (145.894–167.386)	5.345				
	F = 138.297 *p* < 0.001		F = 0.194 *p* = 0.664					
Quality of life						89.333, <0.001, 0.650	6.860, 0.012, 0.125	66.104, <0.001, 0.579
Before the program	36.000 ^a^ (32.268–39.732)	1.856	35.560 ^a^ (31.828–39.292)	1.856	6.860 *			
During the program, week 6	28.200 ^b^ (24.995–31.405)	1.594	35.240 ^a^ (32.035–38.445)	1.594				
End of program, week 12	23.280 ^c^ (20.221–26.339)	1.521	34.560 ^a^ (31.501–37.619)	1.521				
	F = 78.827 *p* < 0.001		F = 24.148 *p* < 0.001					

* *p* < 0.05. Note: Pairwise comparisons were adjusted using Bonferroni correction. CI = confidence interval; SD = standard deviation; η^2^p = partial eta squared. Different superscript letters (a, b, c) within the same group indicate significant Bonferroni-adjusted pairwise differences across time points (*p* < 0.05). Identical superscripts indicate no significant pairwise difference.

## Data Availability

The data presented in this study are available on request from the corresponding author to protect the privacy of the research participants.

## Abbreviations

The following abbreviations are used in this manuscript:

CVI	Chronic Venous Insufficiency disease
rVCSS	The Revised Venous Clinical Severity Score
CIVIQ-20	The Chronic Venous Disease Quality of Life Questionnaire
CEAP	Clinical-Etiology-Anatomy-Pathophysiology
